# Recurrent advanced colonic cancer occurring 11 years after initial endoscopic piecemeal resection: a case report

**DOI:** 10.1186/1471-230X-10-87

**Published:** 2010-08-05

**Authors:** Takayoshi Kishino, Takahisa Matsuda, Taku Sakamoto, Takeshi Nakajima, Hirokazu Taniguchi, Seiichiro Yamamoto, Yutaka Saito

**Affiliations:** 1 Endoscopy Division, National Cancer Center Hospital, Tokyo, Japan; 2 Clinical Laboratory Division, National Cancer Center Hospital, Tokyo, Japan; 3 Colorectal Surgery Division, National Cancer Center Hospital, Tokyo, Japan

## Abstract

**Background:**

The high frequency of local recurrence occurring after endoscopic piecemeal resection (EPMR) for large colorectal tumors is a serious problem. However, almost all of these cases of local recurrence can be detected within 1 year and cured by additional endoscopic resection. We report a rare case of recurrent advanced colonic cancer diagnosed 11 years after initial EPMR treatment.

**Case presentation:**

A 65-year-old male was diagnosed with a sigmoid colon lesion following a routine health check-up. Total colonoscopy revealed a 12 mm type 0-Is lesion in the sigmoid colon, which was diagnosed as an adenoma or intramucosal cancer and treated by EPMR in 1996. The post-resection defect was closed completely using metallic endoclips to avoid delayed bleeding. In 2007, at the third follow up, colonoscopy revealed a 20 mm submucosal tumor (SMT) like recurrence at the site of the previous EPMR. The recurrent lesion was treated by laparoscopic assisted sigmoidectomy with lymph node dissection.

**Conclusion:**

When it is difficult to evaluate the depth and margins of resected tumors following EPMR, it is important that the defect is not closed in order to avoid tumor implantation, missing residual lesions and to enable earlier detection of recurrence. It is crucial that the optimal follow-up protocol for EPMR cases is clarified, particularly how often and for how long they should be followed.

## Background

Endoscopic mucosal resection (EMR) is indicated for the treatment of adenoma and intramucosal or submucosal superficial (SM1: less than 1000 μm from the muscularis mucosa) colorectal cancers because of its minimal invasiveness, negligible risk of lymph-node metastasis[1] and excellent results in term of clinical outcome[2-4]. However, the high frequency of local recurrence after endoscopic piecemeal resection (EPMR) for large colorectal tumors is a serious problem. Previous studies have reported the rate of local recurrence following piecemeal resection to be 25-50%[5,6]. However, almost all cases of local recurrence can be detected within 1 year and cured by additional endoscopic resection, making EPMR an acceptable treatment option. Herein, we report a rare case of recurrent advanced colonic cancer occurring 11 years after initial EPMR treatment.

## Case presentation

The patient was a 65-year-old male with a history of radical prostatectomy for prostate cancer. Following a positive faecal occult blood test, a total colonoscopy was performed at a previous hospital in 1996 and a sigmoid colon lesion was identified. He was referred to our hospital for more precise examination and treatment. Colonoscopy revealed a 12 mm type 0-Is lesion in the sigmoid colon. We diagnosed the lesion as an adenoma or an intramucosal cancer and tried to remove this lesion by *en bloc *EMR. However, as a result the lesion was removed by piecemeal resection (2-pieces). The post-resection defect was closed using metallic endoclips.

**Figure 1:**
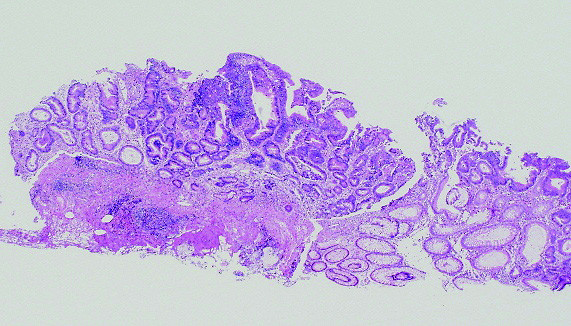
**Histopathological findings of the initial EPMR treatment in 1996 revealing a well differentiated adenocarcinoma with low grade atypia**.

**Figure 2:**
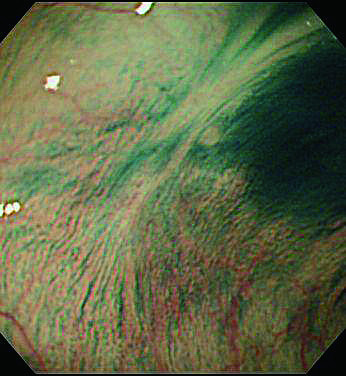
**Follow up colonoscopy 3years after initial EPMR treatment**. The scar was observed at the site of EPMR.

Histopathological examination revealed a well differentiated adenocarcinoma with low grade atypia, and the depth of invasion was intramucosa without lymphovascular invasion, cut end margin negative (Figure 1). We considered the treatment to be a curative resection. Follow up colonoscopy was performed 1 and 3 years after endoscopic resection. The EPMR scar was recognized without any residual or recurrent lesion in the follow up (Figure 2). Follow up colonoscopy was scheduled at 5 years after treatment, but cancelled for personal reasons.

**Figure 3:**
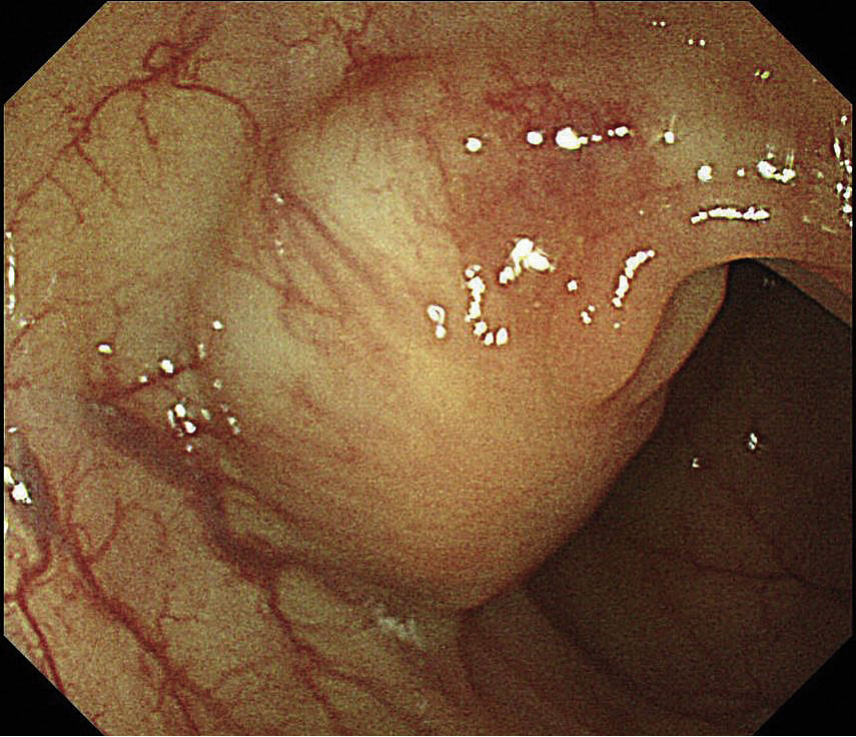
**Follow up colonoscopy in 2007 revealed a protruding subumucosal tumor (SMT) at the initial resection site (a), after indigo carmine dye (b)**.

**Figure 4:**
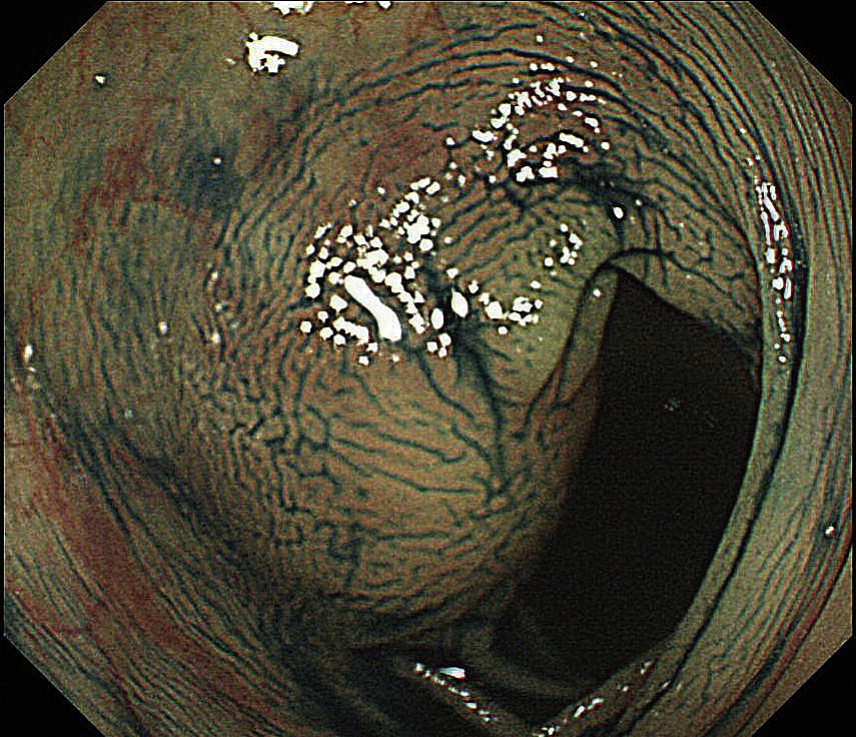
**Follow up colonoscopy in 2007 revealed a protruding subumucosal tumor (SMT) at the initial resection site (a), after indigo carmine dye (b)**.

**Figure 5:**
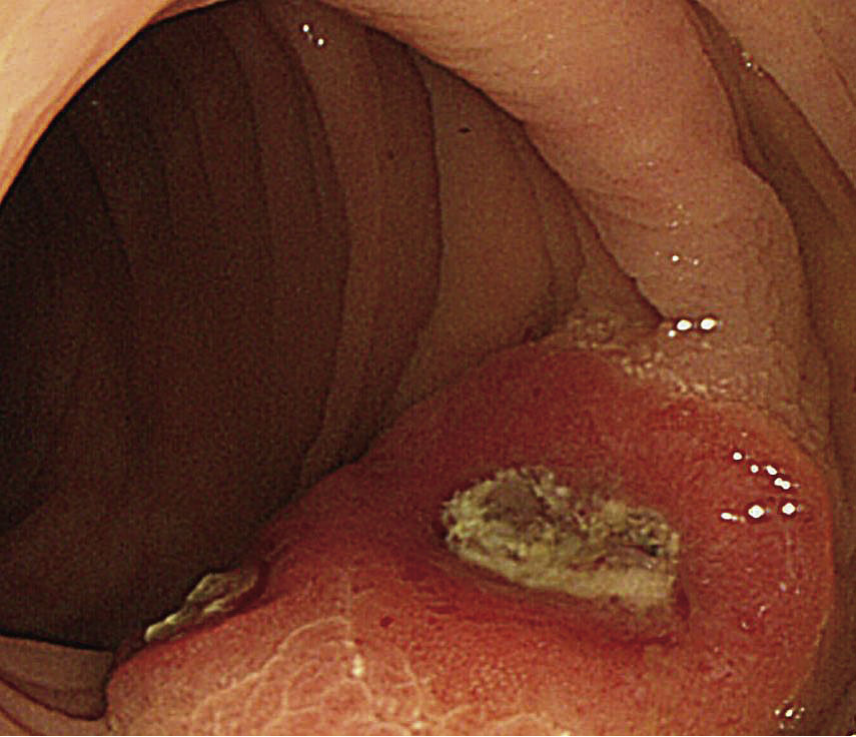
**Colonoscopy revealed that the SMT lesion had grown in size, with a reddish surface pitted with crater-like irregularities (a), after indigo carmine dye (b)**.

**Figure 6:**
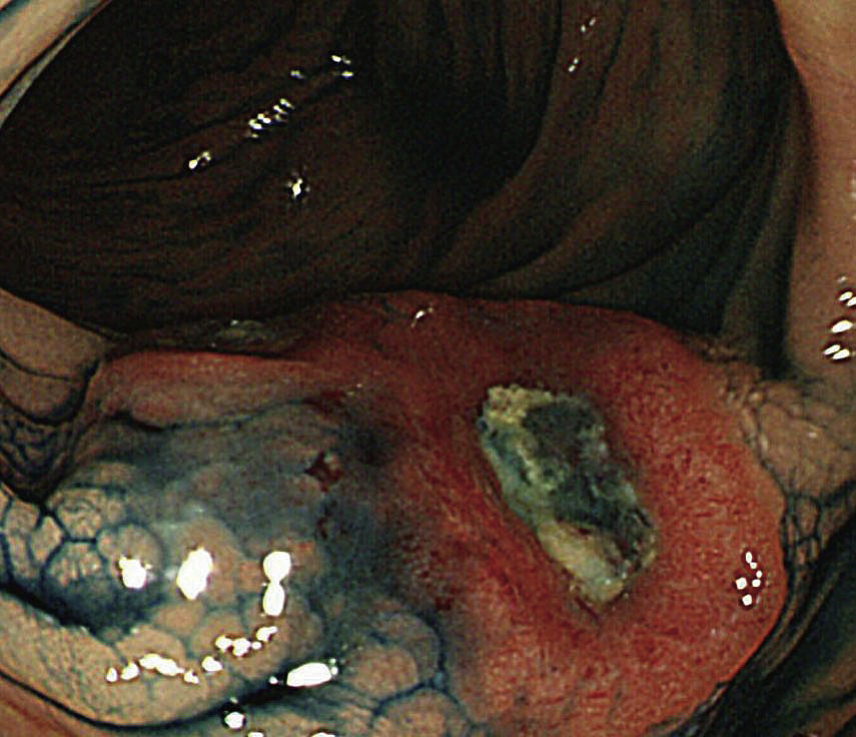
**Colonoscopy revealed that the SMT lesion had grown in size, with a reddish surface pitted with crater-like irregularities (a), after indigo carmine dye (b)**.

**Figure 7:**
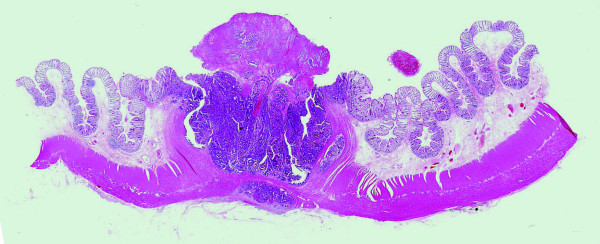
**Histopathological finding of the surgically resected specimen**.

In 2007, the third follow up colonoscopy revealed a protruding submucosal tumor (SMT), 20 mm in size at the site of the 1996 EPMR (Figure 3 and 4). The biopsy specimen from the colonic mucosa did not demonstrate any malignancy. Therefore, we planned a follow up colonoscopy 6 months later. The follow up colonoscopy revealed that the SMT-like lesion had grown to a large size, with a reddish surface pitted with crater-like irregularities (Figure 5 and 6). Histopathological diagnosis confirmed an adenocarcinoma, and a laparoscopic-assisted sigmoidectomy with D3 lymph node resection was performed in 2007. Histopathological analysis of the resected lesion revealed a moderately differentiated adenocarcinoma, and the depth of invasion was subserosa with lymph node metastasis, lymphovascular invasion, venous invasion and perineural invasion (Figure 7).

## Conclusion

In this case, preoperative examination and histopathological findings revealed no evidence of prostate cancer recurrence. The gross configuration of the SMT-like lesion did not support the diagnosis of a primary colonic cancer, and the lesion was diagnosed as a recurrence of the sigmoid colon cancer previously removed by EPMR, with the biopsy specimen very similar to the initial EPMR specimen.

Endoscopic resection for early colorectal cancers has been used throughout the world since the 1970 s and EMR with a submucosal injection technique allows the removal of large colorectal lesions. However, local recurrence frequently occurs after EPMR, which is a serious problem[5,6]. Previous research has indicated that most recurrent tumors after EPMR are found within 7 months and treated with additional endoscopic resection[7]. This present case is very rare due to the following reasons; [1] it is a recurrent advanced cancer following initial treatment of an intramucosal cancer, [2] morphologically SMT-like lesion, [3] late recurrence occurring more than 10 years after EPMR treatment. We speculate that micro-residual lesions were made by the EPMR along the edge of the defect and these were then buried into the submucosa by the endoscopic closure using endoclips. Routine follow up was unable to detect these lesions allowing them to develop into SMT. In addition, the micro-residual lesions developed very slowly because the primary lesion was low grade atypia. According to the Guidelines for Colonoscopy Surveillance after EMR: a consensus update by the US Multi-Society Task Force on Colorectal Cancer and the American Cancer Society, patients with sessile adenomas that are removed piecemeal should be considered for follow up evaluation at shorter intervals (2-6 months) to verify complete removal. Once complete removal has been achieved, subsequent surveillance needs to be individualized based on the judgement of the endoscopist[8]. However, in this case, local recurrence occurred after 11 years, although no residual and no recurrent lesions were identified by the follow up colonoscopy at 1 and 3 years.

Results from this case do not support the need for routine long-term follow up colonoscopy. However in cases where it is difficult to evaluate truly the surgical margin and depth of invasion after EPMR, it is important in order to avoid missing residual lesions and to detect recurrent lesions earlier, that where suitable the defect is not closed and follow up colonoscopy should be performed at appropriate intervals.

## Abbreviations

EPMR: endoscopic piecemeal mucosal resection; ESD: endoscopic submucosal dissection; EMR: endoscopic mucosal resection; SMT: submucosal tumor.

## Consent

Written informed consent was obtained from the patient for publication of this case report and any accompanying images. A copy of the written consent is available for review by the Editor-in-Chief of this journal

## Competing interests

The authors declare that they have no competing interests.

## Authors' contributions

TK collected the data and wrote the report, and was involved in drafting the manuscript. TS was involved in drafting the manuscript. TM revised the manuscript critically for important intellectual content. All authors read and approved the final manuscript.

## Pre-publication history

The pre-publication history for this paper can be accessed here:

http://www.biomedcentral.com/1471-230X/10/87/prepub
